# Aboriginal health research in the remote Kimberley: an exploration of perceptions, attitudes and concerns of stakeholders

**DOI:** 10.1186/s12913-014-0517-1

**Published:** 2014-10-26

**Authors:** Frieda Mc Loughlin, Nyssa T Hadgraft, David Atkinson, Julia V Marley

**Affiliations:** Kimberley Aboriginal Medical Services Council, 12 Napier Terrace, PO Box 1377, Broome, WA 6725 Australia; The Rural Clinical School of Western Australia, The University of Western Australia, 12 Napier Terrace, PO Box 1377, Broome, WA 6725 Australia

**Keywords:** Aboriginal and Torres Strait Islander research, Ethics, Kimberley Aboriginal Health Planning Forum Research Subcommittee, Community consultation, Research processes, Decolonising research

## Abstract

**Background:**

For decades Indigenous peoples have argued for health research reform claiming methods used and results obtained often reflect the exploitative history of colonisation. In 2006 the Kimberley Aboriginal Health Planning Forum (KAHPF) Research Subcommittee (hereafter, the Subcommittee) was formed to improve research processes in the remote Kimberley region of north Western Australia. This paper explores the major perceptions, attitudes and concerns of stakeholders in the Subcommittee.

**Methods:**

Qualitative analysis was carried out on data retrospectively collected from multiple evidentiary sources linked to the Subcommittee i.e. database, documents, interviews, review forms and emails from 1 January 2007 to 31 October 2013.

**Results:**

From 1 January 2007 to 30 June 2013 the Subcommittee received 95 proposals, 57 (60%) driven by researchers based outside the region. Local stakeholders (22 from 12 different Kimberley organisations) raised concerns about 36 (38%) projects, 30 (83%) of which were driven by external researchers. Major concerns of local stakeholders were inadequate community consultation and engagement; burden of research on the region; negative impact of research practices; lack of demonstrable community benefit; and power and control of research. Major themes identified by external stakeholders (25 external researchers who completed the review form) were unanticipated difficulties with consultation processes; barriers to travel; perceiving research as a competing priority for health services and time-consuming ethics processes. External stakeholders also identified strategies for improving research practices in the Kimberley: importance of community support in building good relationships; employing local people; flexibility in research approaches; and importance of allocating sufficient time for consultation and data collection.

**Conclusions:**

Health research in the Kimberley has improved in recent years, however significant problems remain. Prioritising research addressing genuine local needs is essential in closing the gap in Indigenous life expectancy. The long-term aim is for local health service connected researchers to identify priorities, lead, conduct and participate in the majority of local health research. For this to occur, a more radical move involving reconceptualising the research process is needed. Changes to institutional timeframes and funding processes could improve Indigenous and community-based research.

## Background

For decades Aboriginal and Torres Strait Islander peoples have argued for research reform claiming that the methods used and results obtained often just reflect the exploitative history of colonialism and racism in Australia [1-3]. The introduction of ethical guidelines in the 1990s in Australia went some way towards improving ethical conduct in research but many believe they do not challenge, and hence preserve, the entrenched ‘western’ approach to identifying, funding, conducting and controlling health research [2,4].

Historically, health research involving Indigenous peoples has focused on describing the nature and extent of the health problems they face [5,6] and has treated Indigenous peoples as subjects, rather than participants [7,8]. While descriptive research is important for epidemiological surveillance, it does not contribute evidence to expedite positive change or lead to improved health outcomes for Indigenous peoples [6]. Such research is often researcher-driven, rather than community-driven, and therefore does not necessarily address local priorities [9].

The Lowitja Institute is Australia’s only Aboriginal and Torres Strait Islander controlled health research organisation and is guided by the principles of the Indigenous Research Reform Agenda [1,10]. It has developed a research philosophy called the Facilitated Development Approach that identifies research priorities, works with communities to establish research considered desirable and commissions research in partnership with the communities concerned. However much of Aboriginal and Torres Strait Islander health research continues to be based on national research funding (e.g. National Health and Medical Research Council (NHMRC)), which may not reflect local research priorities.

While many Indigenous scholars have written about decolonising research and advocated for Indigenous peoples to control the research agenda, most of this discourse is theoretical [8,11-13]. There are few reports on what actually happens ‘on-the-ground’ in research. What has been published includes case studies of individual projects from the point of view of the external researcher, local Indigenous community and/or health service involved in the research [7,14-18]. Reviews of research ethics committees have focused on how long it takes for approval to be granted, review of letters sent by committees to researchers and case studies of individual projects [19-23].

The remote Kimberley region in the north of Western Australia (WA) covers 423,517 square kilometres, greater than three times the size of England. The state capital, Perth, is over 1600 km away. According to the Australian Bureau of Statistics the total population in 2011 was 34,793, including an Aboriginal population of 13,919 [24]. While Aboriginal people are socioeconomically disadvantaged everywhere in Australia [25], remote Aboriginal people, including most residents of the Kimberley are in the lowest socioeconomic quartile of Aboriginal people [25,26], the most disadvantaged group within the most disadvantaged population in Australia. The health status of Aboriginal people from the Kimberley region continues to lag behind the non-Indigenous population, reflecting the national trend [27,28].

Historically, within the Kimberley region, a large proportion of health research has been developed and carried out by non-Indigenous researchers based outside the region (external researchers) often using approaches that do not reflect Aboriginal perspectives [5]. As a result Kimberley Aboriginal people continue to regard research, particularly research originating outside their communities, as often being exploitative [29].

Most health services in the Kimberley recognise the valuable role research can have in improving health care and health outcomes in the region, and useful research has been carried out [17,30-40]. However, anecdotal reports suggest that many Kimberley health service personnel and Aboriginal communities feel overburdened by health research and perceive little apparent benefit from it. In late 2006 in response to internal pressure within the region, particularly from Aboriginal people, a local research review panel was formed to improve research processes and encourage appropriate research: The Kimberley Aboriginal Health Planning Forum (KAHPF) Research Subcommittee (hereafter, the Subcommittee) [41].

As the number of applications to the Subcommittee increased concerns were raised by Kimberley organisations about the burden of research, and by researchers about the processes to get approval for their projects. The Subcommittee reported this to KAHPF and proposed to review its own activities. Here we describe the major perceptions, attitudes and concerns of local stakeholders connected to the Subcommittee across the Kimberley region and juxtapose them with external stakeholders’ perspectives on the issues they faced conducting research in the Kimberley. The aim of this paper is to give a local perspective and practical examples of how Indigenous communities and their health services expect researchers to approach Indigenous research.

## Methods

### Kimberley approach to research

The Subcommittee is a regional research review panel (not an ethics committee) that aims to foster a coordinated approach to health research and promote research that is meaningful, useful and results in practical change and development within the region [41]. Its aims are to consolidate and strengthen existing networks wherever possible thus enabling maximum benefit to be derived from any research carried out. It also aims to minimise any negative impact of research on the people and health services of the region. There is an agreement between KAHPF member services, which includes all major health service providers in the Kimberley, that all health-related research projects should be submitted to the Subcommittee for consideration.

Subcommittee members are volunteers (hence have limited time) from KAHPF member health services and other Kimberley based organisations involved in research. They include health professionals, researchers, and chief executive officers (CEOs); ensuring Aboriginal representation. Anyone seeking to conduct health research or evaluations in the Kimberley is asked to complete a research proposal form that is circulated to Subcommittee members by email for advice, suggestions and comments. The Subcommittee seeks advice from Kimberley health services and other relevant Kimberley organisations on the potential impact of the research proposed on these services.

All research projects conducted in WA that are focussed on Aboriginal people, or where Aboriginal people are a significant component of the participants, require ethical approval from the Western Australian Aboriginal Health Ethics Committee (WAAHEC) [42] as well as relevant institutional ethics committees (e.g. University, WA Department of Health). Subcommittee endorsement is required by WAAHEC for projects conducted in the Kimberley.

### Stakeholders

Stakeholders in the Subcommittee were defined as any individual or organisation connected to the Subcommittee. For the purpose of this paper, stakeholders were subdivided into two groups: 1) local stakeholders (e.g. members of the KAHPF Research Subcommittee, members of KAHPF, personnel from Kimberley organisations affected by research) and 2) external stakeholders (researchers who were based outside the Kimberley who completed the review form – see below for details).

### Data collection

Data were obtained from multiple evidentiary sources from the Subcommittee. These included documents, terms of reference, the Subcommittee website [41], logs, interviews, letters, review forms, research reports, emails, minutes, newsletters, annual progress reports and draft papers. All data used in the review of the Subcommittee were collected as part of the Subcommittee’s normal procedures, which include auditing its processes.

A researcher independent of the Subcommittee (FML) conducted a retrospective file audit of all the data involving all research project applications submitted to and data processed by the Subcommittee between 1 January 2007 and 31 December 2011. This was updated in late 2013 by another researcher who provided administrative support to the Subcommittee (NH). Each stakeholder was given a code which was used to maintain confidentiality.

In early-2013 the Subcommittee instigated a review process of its operations from the perspective of researchers, which included seeking input about issues faced by researchers associated with conducting research in the Kimberley. The Subcommittee revised its final report form in April 2013 (providing progress reports and a final report is a condition of support from the Subcommittee). This revised form was emailed to past and current researchers titled “Review of Research in the Kimberley” (review form) as part of the review of Subcommittee processes in June 2013. This was in lieu of the usual progress reporting process. Researchers based outside the region associated with 20 projects in progress at the time and 5 completed projects responded to the review. Following this trial the revised final report form was implemented (available from Subcommittee website [41]).

### Data analysis

To provide context projects submitted to the Subcommittee were classified as either driven by the region or by external researchers. It was noted if projects driven by external researchers had research partners based in the region. The number and type of issues arising from project proposals were measured. Next, all raw data were qualitatively analysed and comments that illuminated concepts or revealed an opinion, attitude or insight towards research were noted verbatim into a log. The analysis of data from local stakeholders was conducted by FML. Local stakeholders’ own words were first used to create labels, which were then grouped into codes. A number of key themes were identified and the final stage of data analysis involved finding connections between codes and explanations for these connections to form a coordinated explanatory model [43]. Similarly NH analysed the data from review forms provided by external stakeholders in mid-2013.

An integrated approach to data analysis combined deductive and inductive methods: original coding categories were based on previously identified concepts from the Subcommittee log and review forms and further categories were added as themes emerged from the data in an iterative process [44]. Information that could be used to identify projects or researchers was removed, except where explicit permission was granted.

A draft version of the full report was given to current members of the Subcommittee and KAHPF for comment and to confirm that findings were valid and represented member’s views and experiences. Members of KAHPF, the majority of whom are Aboriginal, endorsed the draft report in the February 2014 meeting as accurately reflecting their lived experience.

A draft of the section on external stakeholders’ responses was sent to researchers who had participated in the review for comment. They were informed that the Subcommittee was planning on making this section of the report public (as a guide for other researchers that could be downloaded from the Subcommittee website [41]). If they had any concerns with this they were provided the opportunity to contact the Subcommittee. Only three researchers commented on the report and no one objected to it being made public. All researchers involved in the Lililwan study [45] (including local Aboriginal community members) agreed to the information about their study being identified in this paper. A modification was made to one quote following a request from a researcher. No other changes were made.

### Ethics approval

WAAHEC was asked to formally assess if conducting an audit of the Subcommittee’s processes could be granted exemption from full ethical review in April 2013, based on the NHMRC guidelines contained in the publication “When does quality assurance in health care require independent review” [46]. WAAHEC determined that the audit processes described did not breach the guidelines and provided exemption from full ethical review in June 2013.

## Results

**Figure 1:**
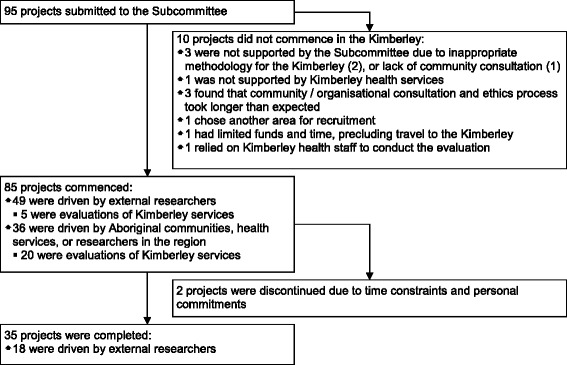
**Flow chart depicting projects submitted to the Kimberley Aboriginal Health Planning Forum Research Subcommittee during 1 January 2007 to 30 June 2013.**

From January 2007 to 30 June 2013, 95 proposed research and evaluation projects were submitted to the Subcommittee (see Figure 1). Discussions about the submitted projects involved 22 local stakeholders (5 Aboriginal stakeholders) from 12 different Kimberley organisations (6 Aboriginal organisations). There was an average of 4.4 responses (52% of available local stakeholders) per project. There was considerable consensus in perceptions of health research in the region among local stakeholders. Of the projects submitted to the Subcommittee 57 (60%) were driven by external researchers and 45 (79%) of these projects had no local research partner. The majority of projects driven by researchers based in the region were internal evaluations (20; 53%), compared to only six (11%) evaluations relating to Kimberley services conducted by external researchers.

If there were no issues with a project the email responses from Subcommittee members were often restricted to “I have no issues with this project” or “I support this project”.

### Local stakeholder concerns

Local stakeholders raised concerns about 36 (38%) projects. Of these 30 (83%) were driven by external researchers and 28 (78%) had no local research partner. The main concerns identified are detailed below.

### Inadequate community consultation and engagement

Local stakeholders acknowledged there had been improvements in the area of community consultation and engagement over the years. They agreed that researchers who took time to build reciprocal, trusting and truly collaborative relationships with communities produced the best research outcomes. However, most felt there were still too many external researchers approaching Kimberley communities looking for letters of support from Aboriginal community members and health services for fully designed and funded project proposals, which left little or no opportunity for meaningful or detailed regional input. Local stakeholders felt that researchers did not seem to be aware of how disrespectful this approach appeared to be and how it destroyed trust.

*Consulting with the sector after the proposal and research design has been developed does not foster a relationship built on respect, trust and cooperation. (S8)*

Local stakeholders were frustrated with external researchers who assumed ‘they knew best’ and were dismissive of local knowledge and advice on regional context, needs and culture.

*I remain somewhat concerned about process and the prospect for genuine partnerships. The invitation of one person to participate in a process to set Aboriginal research agendas for a region with 25% of the Aboriginal population of WA, and for the Kimberley to have had no previous involvement in this process is a concern to me. You need both a strong Aboriginal reference group AND a strong research input from people with research expertise and practical local experience working in health in the region, otherwise you will rightly be accused of having a token consultation process. (S1)*

Local stakeholders acknowledged that one of the reasons the current model was not working was because institutional timeframes and funding schedules often undermined the opportunity for true partnerships and collaboration.

### The burden of health research on the region

A number of Kimberley health service representatives (Aboriginal and non-Indigenous personnel) reported feeling frustrated, overburdened and overwhelmed by the amount of research taking place in the region with little or no perceived benefit arising from it. Local stakeholders considered the relatively high Aboriginal population, beautiful weather (in the dry season) and coastline as major draw cards for conducting research in the Kimberley. Local stakeholders felt that ‘research tourism’ – research that could easily be carried out elsewhere – should be actively discouraged as it was causing community burnout and placing a burden on already overworked health and community services. The Subcommittee project submission form was changed in October 2009 so that external researchers were required to justify why their research needed to be carried out in the Kimberley.

*Although X [government service] wants the best possible services for our clients we are also aware of feedback from [Aboriginal] communities about the drain of information that is gathered from communities with few outcomes in exchange. (S3)*

Local stakeholders noted that many researchers, unintentionally or not, exploited the region’s primary health care resources. For example, researchers would turn up in the region, unaware of the lack of research infrastructure, expecting vehicles, staff and workspaces to be made available to them. Local stakeholders stated that local services in the region were already stretched and were unable to take on extra clinical work generated by some research projects and noted that local services had limited capacity to deliver additional services to remote communities.

*A significant input of resources is required to match the research investment to support building the capacity of local organisations in readiness for the increased referrals. (S2)*

### Negative impact of research practices

Local stakeholders noted that a lot of time and effort was consumed following up on adverse effects of research and believed that processes to deal with these negative impacts needed to be put in place. Local stakeholders also felt it was important to hold researchers accountable for past mistakes to prevent similar errors occurring in the future. Some research projects that involved screening Aboriginal patients for various diseases resulted in work that did not need to be done, as the health services were already aware of the outcomes. Furthermore because some external researchers were unaware of the referral process used in the Kimberley the correct procedures were not followed. This resulted in some Aboriginal patients having significant delays in referral for surgery.

*Months later [after the screening was conducted by external researchers] I saw a patient who said she had been seen by the [specialist] and that she didn't need to see me. This patient needed surgery, however there was no records left that she had been referred. The WACHS [local government health service] system needs a form filled out for referral to [the specialist], however it appears that the [research] team didn't know this. The form is normally filled out by a WACHS nurse and signed by the doctor. Because the researchers did not liaise with WACHS or me the correct procedures were not followed. (S9)*

Local stakeholders believed the burden of a bad project would impact on other potentially more important projects that people in the region may decide needed to be carried out, and ultimately lead to the erosion of public trust and support for research.

*There is a general sense in [X Aboriginal community controlled health service] at least that people may be reaching a state of research saturation for now. Not to mention the recent experience X [Aboriginal CEO] has outlined in another email, highlighting the impact of adverse encounters with researchers on the receptiveness to future research as a whole. (S7)*

Local stakeholders were particularly concerned about the application of pressure to take part in multi-site or national research projects. They felt there should be no expectation that national projects will get a green light in the region, especially if the researchers were not willing to let the region have influence over the research question or design from the outset. They believed that expert driven agendas and a centralised national focus do not always work best for Aboriginal communities or align to the region’s needs and wishes.

*This implies there will be ministerial displeasure if approval is not granted by the [Aboriginal ethics] committee, and could be considered undue application of pressure to accept a research proposal. (S3)*

*The main offenders are actually national projects, often with an Aboriginal reference group, which come to the region and insist that because it is a national project we just have to go along and do as they say. And they almost always do research that is useless to the region or misrepresents us and takes up a lot of time of PHC [primary health care] services! (S4)*

### Demonstrable benefit for the community

Local stakeholders, in particular Aboriginal community members, felt that only useful research with clear and tangible benefits arising from it should be undertaken in the region. The question of ‘whose interests were being served?’ was a central one. Too often local stakeholders felt it was the researchers that benefitted most from research i.e. the advancement of academic, political or professional careers or advantages to institutions in terms of funding under the name of Aboriginal research with few outcomes for Aboriginal communities in exchange.

*There is a sense in your response that your timeframe and resources are more important than the value or benefit of the study for the region. (S5)*

Local stakeholders were frustrated with what they saw as futile research. They raised concerns about the ethics of repeated descriptive research without action when problems were already known and the challenge was finding money and interventions to address these problems. Local stakeholders were disappointed by the indifference shown by governments to their calls to act on previous research outcomes rather than fund more of the same research.

*We are concerned with the lack of built-in intervention in the research process. There is already a considerable body of research that has been undertaken on Kimberley children, the intensive and comprehensive X being a recent example. Following this study, despite presenting clear evidence that Indigenous children carry a heavy burden of both physical and social ill-health, there has been no comprehensive government response to address the matters raised. KAHPF members did not feel confident that your project would, as you suggested, put a sufficiently ‘strong spotlight onto Aboriginal health issues across the country’ to ensure that there was a government response. (S3)*

Local stakeholders believed that the research agenda should be designed to strengthen research capacity building in the region. This would enable the region to realise its vision of Kimberley Aboriginal and non-Indigenous researchers conducting most of their own research in their own communities. They recognised and acknowledged the enormity of this task and noted this was a long-term venture that must be adequately funded.

*We must insist researchers put back into community - An important element of any project in the Kimberley is the training and development of Aboriginal and/or Torres Strait Islander chief investigators and researchers throughout the project period. (S6)*

### Power and control of research in the region

This dominant theme emerged frequently and hinged on the perception of unequal power relations between external researchers and Kimberley Aboriginal communities and organisations. Local stakeholders were frustrated when ‘outsiders’ rather than locals repeatedly got funding to conduct research in the region. Of the 38 projects driven by the region only five received substantial external funding (>$100,000). The majority of Kimberley driven projects, including health service evaluations relied on in-kind contributions.

*I am disappointed about yet another example of groups outside the Kimberley being resourced instead of local providers. (S4)*

Local stakeholders believed the more research carried out by people from the region the more likely it was to be sustainable and responsive to the region’s needs. Failing this, local stakeholders wanted groups that have the capacity and funds to undertake research to come to the region and ‘help us do research with them rather than them doing research on us’.

*We are keen to work closely with research partners but the region needs to have control – not have control vested in any Perth based organisation (even one as benign as X). The way to demonstrate commitment would be to work with us in the Kimberley to help us address regional research priorities rather than us participate in you setting priorities for us. (S1)*

### External stakeholders’ perspectives

The major themes identified from external stakeholders who responded to the Subcommittee’s review (25) are detailed below.

### Unanticipated difficulties with consultation processes

Some external stakeholders thought that the ethics and consultation processes were clearly explained by the Subcommittee and contact people clearly identified. However, many external stakeholders found consultation processes to be more time-consuming than expected. Examples of difficulties encountered by external stakeholders included not knowing who the appropriate contact person was (e.g. CEOs or Chairpersons of local Aboriginal organisations); difficulties making contact with community members in positions of authority; and the length of time needed to build contacts. High staff turnover at Aboriginal community controlled health services was also found to be an issue, as relationships may have been built with an individual who provided support for a project, only to have that person leave the organisation and the consultation process needing to start again. Some external stakeholders suggested that a key list of regional contacts would assist researchers from outside the region.

*It would help if there was available a contacts guide to facilitate the establishment of the essential relationships and to assist in capacity building at all levels. (R7)*

### Conflict between importance of face-to-face visits and barriers to travel

Many external stakeholders recognised the importance of conducting face-to-face visits with local stakeholders and research participants but noted the cost and time barriers to visiting more frequently. Available grant funding generally dictated how frequently they could visit the region. Often, consultation was the component of the research that had to be forgone when funds were limited, or funding was not available until ethics approvals had been granted which limited the ability to consult prior to submitting a proposal.

[We had] *limited funding to travel multiple times to the Kimberley for consultation purposes only. (R1)*

*The main hurdle was not having research funding until I had all the ethical approvals so I could not travel to the Kimberley to consult in person. (R6)*

For one project, available funds and project timeline did not allow for data collection to occur in the Kimberley and hence the project was ultimately carried out elsewhere. The potential negative impacts of barriers to travel on local communities were recognised by some, with one external stakeholder noting that barriers to conducting visits also limited opportunities to mentor local staff involved in the research.

### Research is just one of many competing priorities faced by health services

Many health research projects seek to partner with local health services to collect data. Some external stakeholders recognised the time constraints facing health services and staff, and the need to ensure that these organisations had the capacity and time to participate in research that was more often than not time-consuming, even when the impact on services was expected to be minimal. As noted above, the burden of health research in the region was an important theme arising from local stakeholders.

### Ethics processes are time-consuming

The length of time taken for ethics approvals to be granted was raised by many external stakeholders as a factor that delayed or hindered their project. Some thought the processes needed to be clarified or simplified to assist researchers:

*The timing and processes for ethics approval were not clear. The need to go through multiple, usually tiered bodies adds to the confusion and difficulties. (R4)*

It does not appear that this concern is unique to the Kimberley region; external stakeholders involved in state or nation-wide projects noted that reporting requirements to multiple bodies was particularly time-consuming. A few external stakeholders indicated their support for a streamlining of ethics processes – either reducing the number or aligning the requirements of the separate ethics bodies.

*Standardisation of the requirements for ethical clearance for research involving multiple sites or regions within WA would help facilitate state-wide and national research. (R4)*

### External stakeholders’ strategies that improved the conduct of research in the Kimberley

External stakeholders identified several strategies for improving the conduct of research in the Kimberley. These are detailed below.

### Importance of community support & building good relationships

External stakeholders reported that establishing local contacts and having support from local organisations was crucial in the conduct of their research.

The Lililwan study [45], arising from community need and prioritised by the Aboriginal community, is one example of a project that involved extensive community consultation to ensure that the community was well-informed and supportive of the study before it started and to obtain ‘community consent’. This involved external researchers consulting with community members, community leaders and local and regional service providers, and ensuring they were kept informed throughout the project [47].

*Given the sensitivity of the issue [fetal alcohol spectrum disorders] a major challenge exists in ensuring the community is well-informed and supportive of any study that is considered. The purpose of the visit and this consultation report was to assess the level of support from community members, service providers and governments, and to learn about specific concerns and issues. (R2)*

### Employing local people in research

The benefits to external stakeholders and Aboriginal communities arising from employing local people on projects also emerged as a theme. Projects that employed local Aboriginal people as research assistants or interpreters found this assisted with data collection – particularly in locating or following up participants – and respecting local protocols.

*The local Aboriginal community navigators were instrumental in locating parents/carers and organising meeting times. (R2)*

*These processes were made easier as we had a local Aboriginal woman, who was able to arrange visits. (R3)*

However, projects submitted to the Subcommittee rarely employed local Aboriginal people and when this occurred it was generally short-term and casual with limited hours.

Local advisory groups were also used by some external stakeholders, who found these to be valuable in providing advice, suggestions and cultural guidance.

*[The Advisory panel] assisted us in using local knowledge to refine our interview questions so that we could better target the issues that were most important to young Indigenous men in Broome. (R5)*

### Being flexible in approaches to research

While some external stakeholders appeared surprised by the challenges they faced in conducting research in the region, others where more mindful at the beginning of their research that a flexible approach was essential when conducting research with Aboriginal communities in the Kimberley.

*Timing of consultations was important, the ability to be flexible and conduct interviews at different locations (places that were convenient for the participants) was important. (R4)*

*High staff turnover, sudden change of arrangements because of changing priorities or members of the communities leaving for cultural reason were not seen as major hurdles but rather recognized as part of working in Aboriginal communities. (R1)*

### Importance of allowing sufficient time for consultation and data collection

Allowing ample time for data collection and community consultation was noted to be important.

*The primary challenge was to recognise early on that many Kimberley based stakeholders are very busy. In turn this meant developing communication strategies…that allowed enough time for sending out initial emails of introduction and then following these up with phone calls in order to connect and discuss relevant issues relating to [the study]. (R8)*

*Another challenge is simply managing the distances between locations and working out the most effective (and cost effective) ways to visit services and meet regularly with staff face to face. The solution to this is simply good planning and synchronising visits. (R8)*

*Visits of 1–2 weeks duration in each area helped to overcome these issues and achieve the desired number of participants. (R1)*

## Discussion

Our case study of health research in the Kimberley shows that although there has been some transformation in recent years (such as a trend towards more meaningful consultation, collaboration and an increased participatory focus to research methodologies [17,30-35,37,38,47]), research involving Aboriginal people in the Kimberley, as with Indigenous people in other areas of the world, is still dominated by ‘white’ research practices [8,48,49].

Significantly our case study brings together ‘on-the-ground’ reality in terms of the research process from both the perspective of a local research committee (endeavouring to manage the system) and external researchers (attempting to utilise it) across a large geographical area over several years. Our case study is unique in that it provides a practical example of how local communities and health services can ‘talk back’ to researchers and provide a direct advocacy role in decolonising research.

Local stakeholders raised concerns with over half of the projects driven by researchers not based in the Kimberley, compared to only 16% of the Kimberley driven projects. It is possible that some of this was due to local stakeholders being more likely to scrutinise external researchers. However it is much more probable that most of the difference was due to most external researchers approaching local Aboriginal communities and health services after the project has been fully developed, with no local consultation as to the appropriateness of the project or the chosen methodology. In contrast, Kimberley driven projects are generally embedded in local health services and include health service providers as core members of the research team [31-35,37,47]. Kimberley based researchers are normally required to undertake relevant consultation prior to approval being granted by their host organisation.

Despite successive ethical guidelines and Government reviews over the last few decades advocating the need for greater Indigenous control over the research process [50-55] there has been little movement on the ground [56]. The central goal of Indigenous people controlling health research about their people has not been realised [57]. Although ethical guidelines are in place to ensure adequate community consultation occurs, in practice, it is ultimately the researchers retaining the power and the recognised authority in the research process [52]. Most research in the Kimberley was driven by non-Indigenous researchers who were not based in the Kimberley. There was pressure placed on local communities to accept national research projects even if they were not in the best interest of the community.

One of the reasons the current model is not working is because institutional timeframes and funding schedules undermine the aims of ethical guidelines and perpetuate the dominance of non-Indigenous control over the research agenda. Closing the gap in Aboriginal disadvantage in Australia and beyond, will require significant long-term and collaborative effort in health research. While the approach taken by the Lowitja Institute has proved highly successful [10], Kimberley Aboriginal communities have not been involved in this process and there is no evidence to suggest that the achievement of a national approach to the issues raised in this paper is on the horizon [57].

A strength of our study was that a researcher who was independent of the Subcommittee assessed all documentation relating to the Subcommittee during 2007–2011. However, the assessment of review forms filled out by external stakeholders was carried out by a researcher who provided administrative support to the Subcommittee, which could be viewed as a limitation. The opportunity for external stakeholders to comment on the section of the paper related to their comments helped reduce the risks of misrepresentation and as only minor edits to one quote by an external stakeholder were requested, it seems likely that the external stakeholders did not feel misrepresented.

Another limitation is that projects with no issues rarely elicited detailed responses, probably due to high workloads of Subcommittee members, and thus this paper concentrates on the concerns raised by local stakeholders about submitted projects. The other issues focused on were those raised by external stakeholders who responded to the review. The responses shared common themes about issues and barriers faced by them. These findings are expected to be useful to researchers seeking to undertake health research in the Kimberley as well as Indigenous and community-based research more generally in Australia and elsewhere in the world. Similar research to explore and elicit views from community members and Aboriginal researchers would expand our results.

The findings from this review are being used to guide improvements to the Subcommittee’s processes, including the advice it offers to new researchers and streamlining ethics processes for Kimberley health research. Other research that is planned (subject to funding) is further mapping and assessing the impact of the research carried out in the region on policy and practice and investigating the extent to which building of research capacity of individuals and organisations in the Kimberley has occurred.

While this case study has highlighted some negative aspects of research conducted in the region, during this period there were also a number of valuable projects carried out that had short to medium term positive effects on health services. These include the Lililwan project [45], which has raised awareness and increased services for fetal alcohol spectrum disorders and a pandemic influenza study [58], which led to a change in the national pandemic action plan, and capacity building and employment for two local Aboriginal people [17].

## Conclusion

Funding and prioritisation of community-based and Indigenous research is still largely controlled by people far removed in geography and/or culture from the people being ‘researched’. Our case study provides a practical example of how Indigenous communities and their health services can begin to take control of research in their region. Local stakeholders need to develop strategies to challenge the status quo, to ensure local people develop the necessary capacity to execute and direct health research, and to limit the extent of external control over local research processes.

## Abbreviations

CEOs: Chief executive officers
KAHPF: Kimberley Aboriginal Health Planning Forum
NHMRC: National Health and Medical Research Council
WA: Western Australia
